# MR thermometry-guided ultrasound hyperthermia of user-defined regions using the ExAblate prostate ablation array

**DOI:** 10.1186/s40349-018-0115-5

**Published:** 2018-08-13

**Authors:** Eugene Ozhinsky, Vasant A. Salgaonkar, Chris J. Diederich, Viola Rieke

**Affiliations:** 1 Department of Radiology and Biomedical Imaging, University of California San Francisco, 185 Berry Street, Suite 350, Box 0946, San Francisco, CA 94107 USA; 2 Department of Radiation Oncology, University of California San Francisco, 2340 Sutter Street, S331, Box 1708, San Francisco, CA 94115 USA

**Keywords:** Hyperthermia, MR-guided, MR thermometry, Prostate

## Abstract

**Background:**

Hyperthermia therapy (HT) has shown to be an effective adjuvant to radiation, chemotherapy, and immunotherapy. In order to be safe and effective, delivery of HT requires maintenance of target tissue temperature within a narrow range (40–44 °C) for 30–60 min, which necessitates conformal heat delivery and accurate temperature monitoring. The goal of this project was to develop an MR thermometry-guided hyperthermia delivery platform based upon the ExAblate prostate array that would achieve uniform stable heating over large volumes within the prostate, while allowing the user to precisely control the power deposition patterns and shape of the region of treatment (ROT).

**Methods:**

The HT platform incorporates an accelerated multi-slice real time MR thermometry pulse sequence and reconstruction pipeline. Temperature uniformity over a large contiguous area was achieved by multi-point temperature sampling with multi-focal feedback power control. The hyperthermia delivery system was based on an InSightec ExAblate 2100 prostate focused ultrasound ablation system, and HeartVista’s RTHawk real-time MRI system integrated with a 3 T MRI scanner. The integrated system was evaluated in experiments with a tissue-mimicking phantom for prolonged exposures with a target temperature increase of 7 °C from baseline.

**Results:**

Five various shapes of the region of treatment, defined on a 5 × 5 grid (35 × 35 mm, 11–25 focal spots per shape), were implemented to evaluate the performance of the system. MR temperature images, acquired after steady state was reached, showed different patterns of heating that closely matched the prescribed regions. Temperature uncertainty of the thermometry acquisition was 0.5 °C. The time to reach the target temperature (2:58–7:44 min) depended on the chosen ROT shape and on the distance from transducer to focal plane. Pre-cooling with circulating water helped to reduce near-field heating.

**Conclusions:**

We have implemented a real-time MR thermometry-guided system for hyperthermia delivery within user-defined regions with the ExAblate prostate array and evaluated it in phantom experiments for different shapes and focal depths. Our results demonstrate the feasibility of using a commercially available endorectal FUS transducer to perform spatially-conformal hyperthermia therapy and could lead to a new set of exciting applications for these devices.

## Background

Hyperthermia therapy (HT) involves raising the temperature of the target tissue to 40–44 °C for 30–60 min. It has shown promise as an adjuvant to radiation, chemotherapy, and immunotherapy for the treatment of cancer [1–3]. Recent reviews clearly indicate that HT in conjunction with radiation and/or chemotherapy can significantly improve local control and overall survival for deep and superficial tumors [4, 5]. In pelvic cancers, randomized clinical trials determined that HT added to radiation therapy can improve treatment outcomes for advanced and recurrent cervical cancer [6–10], prostate cancer [11, 12], and persistent pelvic tumors [13]. HT augmented chemotherapy has been proven to increase overall survival in patients with soft tissue sarcoma [14–16]. Clinical studies have demonstrated feasibility and efficacy of prostate hyperthermia with endorectal ultrasound applicators in combination with radiation therapy [11, 17, 18].

In all these applications of hyperthermia therapy, accurate thermal dosimetry and heating conformability are essential for good response. In order to be safe and effective, HT requires maintenance of target tissue temperature within a narrow range (40–44 °C), avoidance of excessive temperatures, which are associated with complications. Thermal dose, measured in CEM43°C (a cumulative equivalent number of minutes at 43 °C) has been used to quantify the amount of thermal exposure during the treatment. For HT, depending upon the heating regimen, a minimum delivered thermal dose threshold of approximately 5–10 CEM43°C T90 (90% of measured points exceeding this value) must be reached [10, 19–21]. This necessitates methods for precise heat delivery and accurate temperature monitoring.

MR imaging techniques, such as proton resonance frequency shift imaging (PRFS) [22], are capable of non-invasive temperature monitoring of hyperthermia and thermal ablation procedures. MR thermometry is commonly used to monitor high temperature ablation of deep tissues with techniques, such as MR-guided focused ultrasound (MRgFUS) therapy, where focused high intensity ultrasonic energy is delivered from an extracorporeal transducer in repeated short sonications. MR thermometry has been successfully utilized for monitoring [23, 24] and control [25] of HT treatments with radiofrequency applicators for heating deep tumors within the pelvis and trunk. MR-guided ultrasound hyperthermia (MRgHT), which uses similar technologies to MRgFUS for prolonged heating at sub-lethal temperatures, has been demonstrated in research studies, but is yet to be available clinically. A multi-element ultrasound array for hyperthermia delivery to the prostate has been investigated for control of hyperthermia and drug delivery under MRI guidance [26, 27]. Staruch et al. developed a focused ultrasound system that performed HT delivery with mechanical scanning of a custom-made ultrasound transducer and closed-loop temperature feedback control [28].

There is interest in utilizing commercially available MRgFUS systems, designed and approved for thermal ablation, for delivery of prolonged moderate heating by modifying their control systems. Using the Philips Sonalleve platform with sparse phased array in-table transducers, Partanen and colleagues implemented a number of approaches for MR-guided HT, such as single focus, simultaneous multi-focus and rapid sweeping trajectories [29], along with separate heat-up and heat maintaining subtrajectories with MR thermometry feedback [30]. The MRgHT system developed by Tillander, et al. heated smaller cells (18–58 mm) by electronic steering of the beam in concentric circles and mechanical transducer steering to cover larger areas [31]. We have previously demonstrated the use of a commercial focused ultrasound (FUS) system to achieve protracted hyperthermia delivery HT [32] using a single region of interest (ROI) and proportional-integral feedback power control. These in vivo studies were useful to establish the feasibility of generating enough sustained power output with the ExAblate prostate array (InSightec, Haifa, Israel) to deliver hyperthermia. It became clear, however, that in order to offer more adjustable and precise delivery of uniform HT to organs such as prostate, there is a need for heating with multi-point thermometry feedback and spatial beam control that could be sustained over long durations.

The objectives of this project were to develop and evaluate an integrated software platform to drive a commercial endorectal prostate ablation array for MR thermometry-guided HT delivery. A technique for conformal power deposition, based upon multi-point temperature feedback power control and up to 25 pre-set focal patterns, has been devised for improved spatial control of energy delivery within a region of treatment (ROT). This multi-focal approach required MR thermometry imaging with high frame rate and low processing delay. Therefore, an accelerated multi-slice real-time MR thermometry pulse sequence and reconstruction pipeline specific for guidance, monitoring, and control of the prostate array were incorporated. This integrated system was designed to achieve uniform stable heating over large volumes, while allowing the user to precisely control power deposition patterns and the shape of the region of treatment (ROT).

## Methods

### System overview

**Fig. 1:**
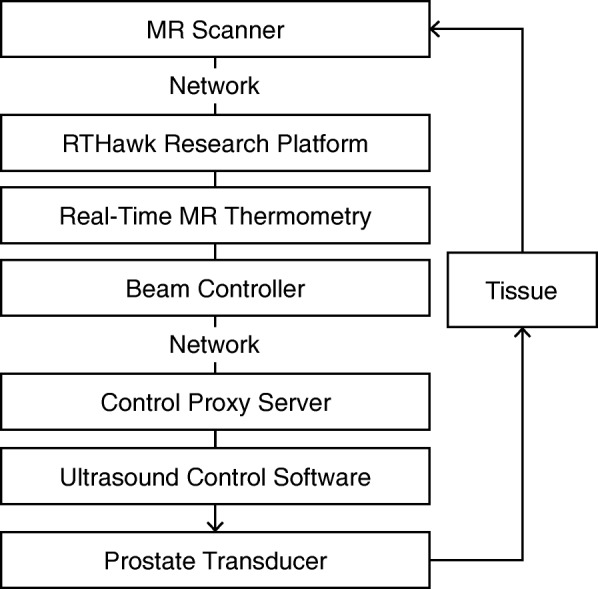
Control flow diagram of the MR thermometry-based hyperthermia delivery system. The RTHawk research platform acquires and reconstructs the real-time MRI data. The real-time MR thermometry application generates temperature maps. The beam controller samples temperature values within a grid of ROIs and controls the focus and power output of the ultrasound beam, ensuring constant and uniform heating within the region of treatment. The control proxy server relays commands from the beam controller to the manufacturer’s ultrasound control software

The hyperthermia delivery system was based on an ExAblate 2100 prostate focused ultrasound system, and an RTHawk real-time MRI system (HeartVista, Inc., Menlo Park, CA) integrated with a 3 T MRI scanner (GE Healthcare, Waukesha, WI). The ExAblate endorectal array (23 × 40 mm size) operated at a center frequency of 2.3 MHz with approximately 1000 independently controlled elements [33, 34]. The system is capable of electronic focusing and rapid switching between up to 32 preset focal patterns. Acoustic coupling and cooling were performed with chilled water (12–14 °C), circulating inside a balloon and enveloping the transducer array. Imaging was performed with the body coil. As the ExAblate system was designed for tumor ablation with short focused sonications, it required significant operational modifications to enable prolonged controlled heating of large areas, necessary for HT delivery. For this project, in order to control the prostate array, an integrated software was developed that included a feedback control loop, an accelerated MR thermometry acquisition and reconstruction application, a beam controller module, and an interface to the manufacturer’s hardware control system (Fig. 1).

### Accelerated real-time MR thermometry

**Fig. 2:**
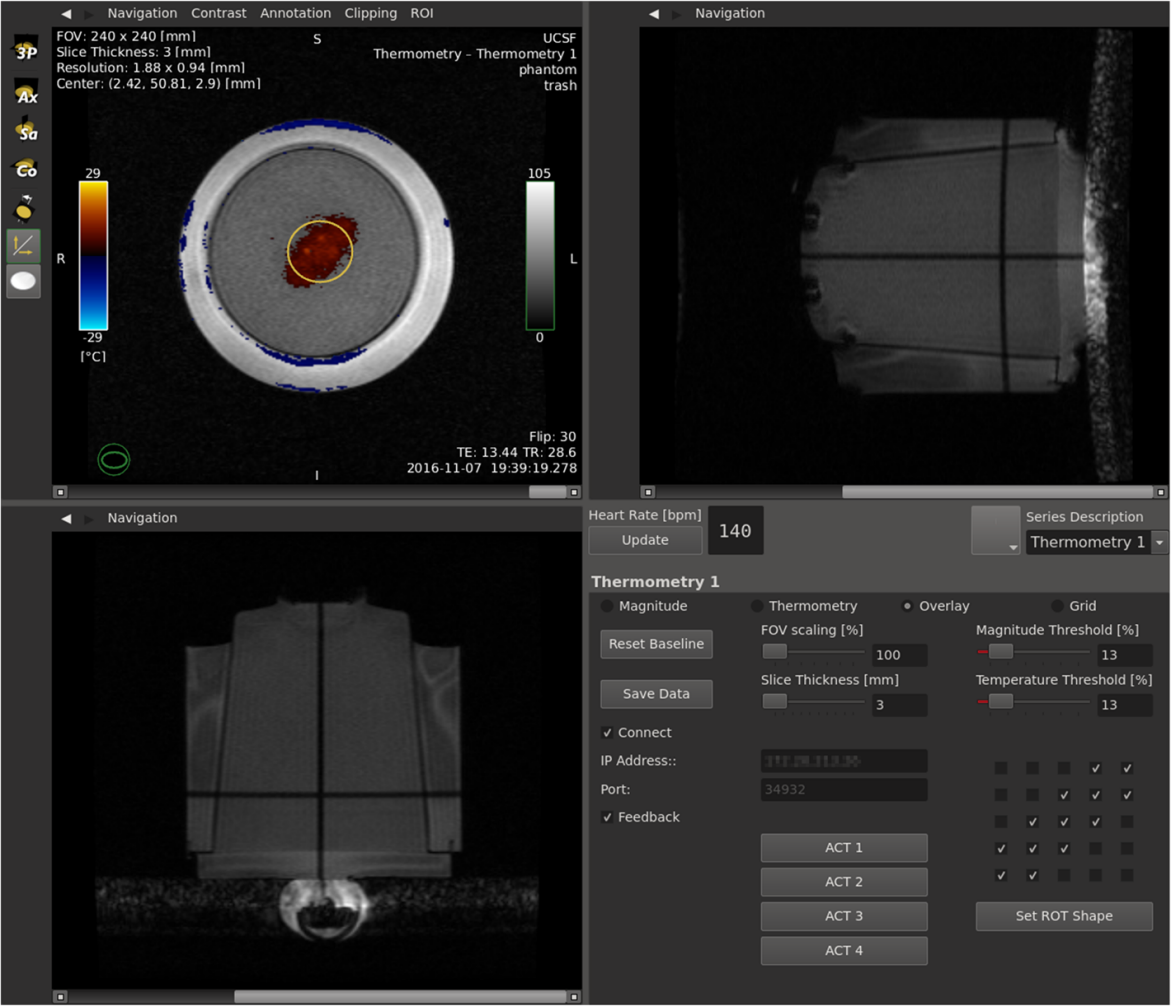
User interface of the real-time MR thermometry application, showing a temperature map overlaid on top of the coronal magnitude image (top-left), sagittal and axial navigator images with the phantom in a water container on top of the focused ultrasound transducer (top-right, bottom-left), and control panel (bottom-right)

An accelerated real-time MRgHT application (Fig. 2) was developed on the RTHawk platform. It allowed continuous acquisition and reconstruction of temperature maps with minimal latency using a spoiled gradient echo (SPGR) pulse sequence, a real-time proton resonance frequency shift (PRFS) thermometry reconstruction pipeline, and a custom interface for data visualization and prescription.

**Fig. 3:**
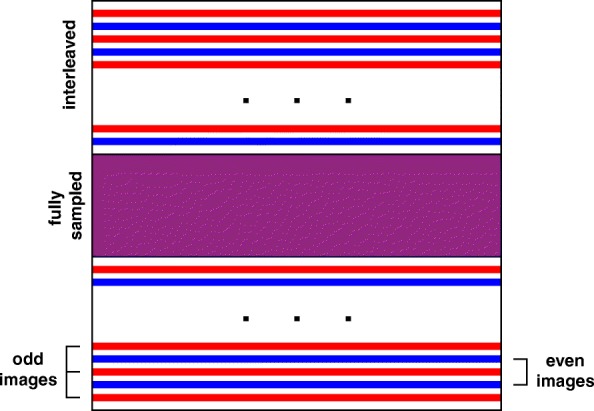
Diagram of the shared k-space with keyhole accelerated readout for an interleaving factor of 2. Odd lines in the periphery of the k-space (red) are updated on odd frames, even lines on even frames (blue), and central lines on every frame (purple), achieving 1.6× acceleration

The pulse sequence implemented a shared k-space with keyhole acceleration technique [35], where the central part of k-space was fully sampled and acquired for every imaging frame. Peripheral lines of k-space were split into alternating sets that were updated less often (Fig. 3). Raw data was transmitted over a local area network to the RTHawk workstation and reconstructed in real-time. The system supported interleaved acquisition of multi-planar MR thermometry data with variable frame rates.

### Beam controller

Temperature values within each MR thermometry frame were sampled within the cells of a 5 × 5 grid that was placed over the region of treatment in the control imaging plane perpendicular to the US beam direction. The FUS system was set up with a library of 25 pre-generated single focus patterns that matched the cells of the grid, covering in the case of our experiments a 35 × 35 mm area. The size of this area and localization of the focal points would be configured based on patient anatomy before each treatment. To control the shape of the region, individual cells of the grid can be turned on and off in the application user interface. This allows for the heated area to conform to the shape of the tumor in the HT treatment. In this study, five distinct shapes of the region of treatment within this grid were implemented to evaluate the performance of the system: square, elliptical, diagonal, triangular, and rhombic.

The controller module determined the next focal point and power level, which were communicated over the network to the FUS system via a Control Proxy Server application (Fig. 1). To achieve uniform heating of the desired area the beam controller directed the beam towards the cell with the lowest temperature within the grid. Based on the measured mean temperature *(T)* within this cell, the controller modulated the power output of the transducer. To reach the target temperature in the shortest amount of time, the output was set to maximum power selected for the treatment *(P = P*_*max*_*)* during the heat up stage *(T < T*_*target*_ *- 0.5 °C)*. Approaching the target temperature *(T*_*target*_ *- 0.5 °C < T < T*_*target*_*)* a proportional controller was used *(P = P*_*max*_ * (T_*target*_ *- T) / 0.5°)*. At or above the target temperature *(T ≥ T*_*target*_*)*, the power output was set to zero *(P = 0)*. Also, as a safety precaution, the power output was set to zero if no thermometry images in the control plane are received in 3 s.

### Experimental setup

The integrated hyperthermia control system was evaluated in experiments with a tissue-mimicking phantom (InSightec) with thermal and acoustic properties of non-perfused tissue (heat conductivity ~ 0.38 W/(m*°C), density = 1.05 g/cc, heat capacity 4.2 J/(g*K)). Prolonged exposures with a target temperature increase of 7 °C from baseline were prescribed. The phantom was placed in a container with a bottom mylar membrane, submerged in water at ambient temperature of approximately 20 °C and positioned over the ultrasound transducer. Several MRgHT experiments with different ROT shapes and sonications parameters were performed as described in Table 1.

**Table 1:** Summary of experiments performed with the system

Experiment	ROT shapes	Near field monitoring	Depth	Acoustic power	Pre-cooling time (min)	Time to target temperature (min)	Time at steady state (min)
1	square	No	60 mm	26.8 W	–	7:44	5:04
	elliptical					3:24	4:19
	diagonal					6:59	5:26
	triangular					6:39	9:34
2	rhombic	Yes	60 mm	20 W	–	6:11	5:29
3	rhombic	Yes	35 mm	20 W	–	2:50	8:18
4	rhombic	Yes	35 mm	20 W	28	2:58	8:13

The purpose of experiment 1 was to evaluate the ability to achieve heating patterns that matched the prescribed ROT shapes in the coronal plane. The MR thermometry imaging slice used for beam control was placed at the level of the ROT orthogonal to the beam axis. Images (256 × 128 matrix, TE = 13 ms, TR = 29 ms, FOV = 24 cm, readout bandwidth = 12.5 kHz, slice thickness = 3 mm) were acquired with 32 lines of central k-space at every frame and an interleave factor of 2 for peripheral k-space, resulting in a 1.6× acceleration over the fully-sampled acquisition. Temperature was sampled within a 35 × 35 mm grid ROI (5 × 5 cells, 7 × 7 mm each).

In experiments 2 and 3, the focal plane was placed at two different levels (60 and 35 mm) and near-field and far-field heating during the treatment was monitored in an axial imaging slice, positioned along the beam axis. The control and monitoring frames were interleaved with one monitoring frame acquired after eight control frames (every 22 s).

Experiment 4 investigated the effect of pre-cooling the near-field area before treatment. Chilled water (12 °C) was circulated in the balloon surrounding the transducer for 28 min before hyperthermia started. MR thermometry measurements were verified with a fiber-optic probe, embedded in the phantom outside the beam path.

## Results

**Fig. 4:**
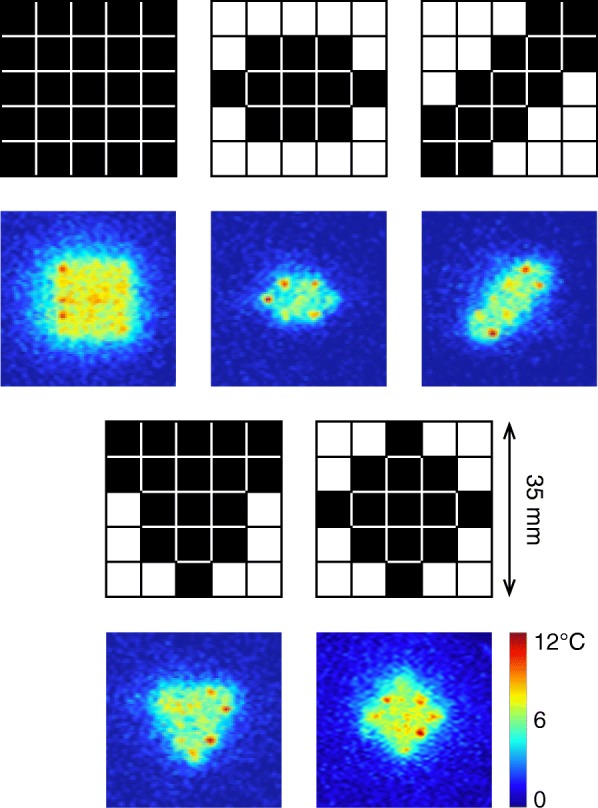
Examples of prescribed regions of treatment (square, elliptical, diagonal, triangular, and rhombic), and corresponding MR thermometry images, acquired during the heating in experiments 1 and 3, showing the flexibility to control the shape of the heated area. Peak temperatures corresponded to recently active focal points

**Fig. 5:**
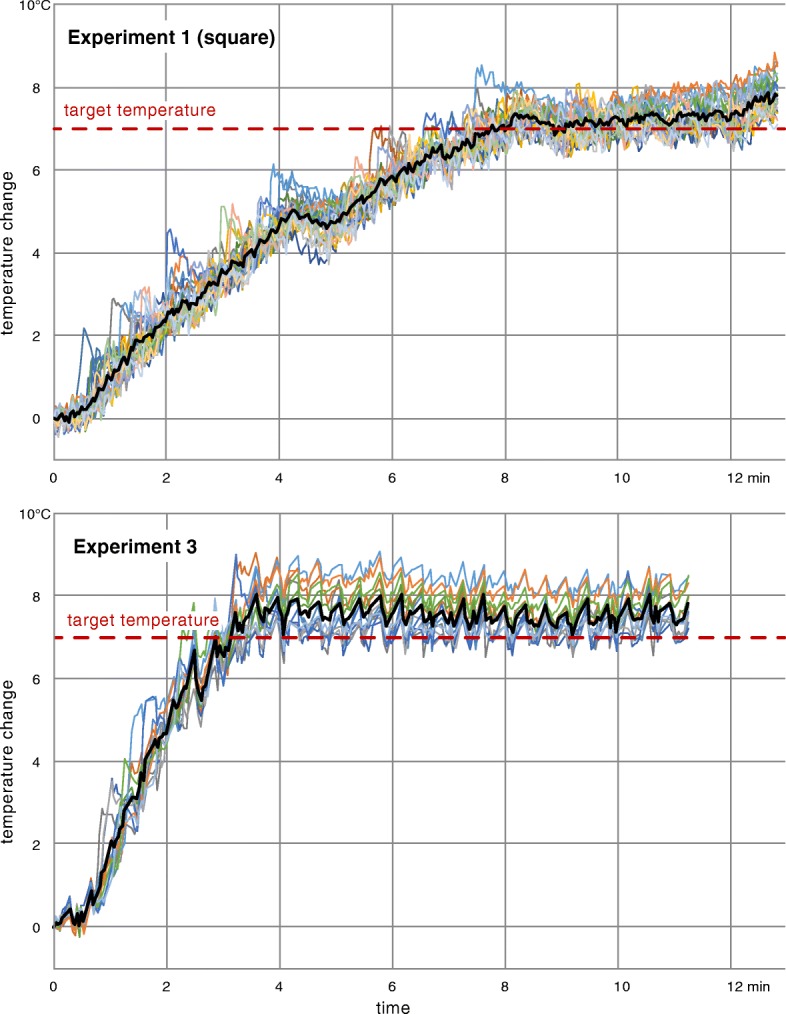
Plots of mean temperature values within cells of the ROT (each cell represented by a different color) and mean temperature of the whole ROT (black) for experiment 1 (top, 60 mm focal depth, square pattern) and experiment 3 (bottom, 35 mm heating depth, rhombic pattern). Brief power output interruption due to the limits on continuous sonication can be seen in the top figure after the 4 min mark and in the bottom figure after the 2 min mark

Multiple heating trials were performed to evaluate system performance, as summarized in Table 1. The table shows the time it took to reach steady state and the duration of steady state heating in each experiment. MR temperature images from experiment 1 and 3, acquired after steady state was reached, showed different patterns of heating that closely matched the prescribed regions (Fig. 4). Temperature uncertainty of the thermometry acquisition was 0.5 °C. Figure 5 shows temperature plots for the cells of ROTs in experiments 1 and 3. The average temperature within the ROT increased until it leveled off near the target temperature. There were brief interruptions in power output of the transducer due to software limits on continuous sonication, as can be seen in Fig. 5 (top) after the 4-min mark.

The time to reach the target temperature depended on the chosen ROT shape and on the distance from the transducer to the focal plane, as seen in Table 1 and Fig. 5. For the square heating pattern in experiment 1, the whole region of treatment reached the target temperature after around 7 min. The elliptical ROT, covering a smaller area, reached steady state in half that time. The rhombic ROT with focal plane at 60 mm from the transducer (experiment 2) took around 6 min to reach steady state. Reducing this distance to 35 mm (experiment 3) reduced the time to around 3 min.

After the ROT reached the target temperature, stable heating was maintained for 4–9 min (Table 1). Figure 5 shows the temperature of the cells of the ROT in experiment 1 (square pattern, mean: 7.3 °C, range: 6.2–8.8 °C) and in experiment 3 (rhombic pattern, mean: 7.4 °C, range: 5.7–9.1 °C). The cells at the periphery of the ROT experienced higher fluctuations in temperature due to heat dissipation into the surrounding area, whereas the inner cells had less variation. Data from experiment 3 showed higher fluctuation of mean temperature than that of experiment 1. This was caused by increased rate of heating due to the focal plane being closer to the transducer and by brief sonication pauses while the system was acquiring monitoring thermometry images (every 22 s). There were image artifacts in one coronal thermometry frame following each axial monitoring frame due to spin steady state effects. These frames were excluded from analysis.

In order to reach the target temperature within a large region in a reasonable amount of time, the transducer initially operated at high power (20-27 W) that was reduced after approaching the target temperature. Fast imaging was important to minimize the risk of overheating the tissue at the beam focus during the intervals between focus changes. The shared k-space with keyhole acceleration technique allowed to improve the acquisition frame rate from 3.7 to 2.3 s update time per frame and provided for a smoother heating curve.

Experiments 2 and 3 investigated pre-focal heating when sonicating at two different depths with near-field monitoring in the transverse plane. As expected, while it was possible to achieve and control target temperature increase at the depth of 60 mm from the transducer (experiment 2), this depth was beyond the effective focusing range of this phased array in soft tissue, which resulted in excessive near-field heating of up to + 22 °C. In vivo, this would have caused tissue ablation. In experiment 3 the target plane was set at 35 mm from the transducer. This decreased the temperature elevation in the near-field to + 9 − + 12 °C from baseline.

**Fig. 6:**
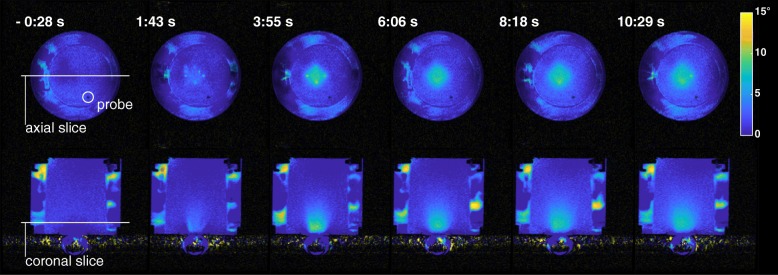
Coronal MR thermometry images used for beam control (upper row) from different time points during the experiment, and axial images (lower row) used for near-field monitoring, acquired during experiment 4. The labels show the time from the heating start (focused ultrasound power on). Flow artifacts within the water surrounding the phantom and in the balloon surrounding the transducer did not affect image quality within the phantom

In experiment 4 (Fig. 6), pre-cooling with circulating water has decreased the temperature near the bottom of the phantom to − 13 °C from baseline, and it remained at or below baseline for the duration of the heating. The cooled area extended 16 mm from the bottom surface of the phantom during the cooling stage and around 3 mm during heating. The tissue in the beam path further from the surface (3–25 mm) experienced heating of up to + 9 − + 10 °C from baseline. The temperature in the phantom outside the beam path increased by around 1 °C during heating due to thermal diffusion from the region of treatment. The fiber-optic probe measurements showed absolute temperature increase from 19.3 to 20.3 °C during the same period.

## Discussion

There have been recent developments in MRgHT using existing commercial ablation systems that underscore the promise of this treatment modality. Previous approaches with the Sonalleve MR-HIFU system (Philips, Profound Medical) utilized treatment cells composed of concentric sub-trajectories [29, 31]. Our previous development for an endorectal phased array transducer featured diverging or fixed beams with power modulation [32]. This approach controlled the mean temperature of the ROT, but it allowed little control of temperature distribution within the ROT. The approach described in this paper enables conformal heating to precisely control the shape of the temperature profile within a selected area. Electronic steering of the beam based on real-time MR thermometry data provided for uniform temperature within the ROT defined on the control plane. Multi-planar thermometry allowed to monitor near- and far-field heating.

The HT system, described in this paper was designed to provide precise conformal heating of a 2D ROT perpendicular to the beam direction. For precise 3D ROT shaping, a 3D set of sampling locations with corresponding focal positions could be used, with additional temperature images obtained in a multi-slice or multi-planar acquisition.

The time to reach the target temperature depended on the chosen ROT shape, and on the distance from the transducer to the focal plane. Even for the largest ROT area (35 × 35 mm square) and large distance (60 mm), the target temperature was reached in about 7 min, which would be sufficient for hyperthermia treatments without requiring longer procedure times. In our experiments, stable heating was maintained for up to 9 min due to time constraints. There were no technical limits that would prevent the system from maintaining HT for longer periods of time (30–60 min).

The power control scheme implemented in this paper used several levels: maximum power selected for the treatment during heat-up, a variable power setting near the target temperature, to zero power once the target temperature was reached. This control scheme was designed to reach the target temperature in the shortest time, while avoiding damaging temperatures at the foci. Unlike the diverging beam approach, which avoids hot spots but lacks a conformal treatment area, the electronically steered focused beam used here carries the risk of overheating the tissue at and around the individual foci. Rapid steering between foci and accurate monitoring is therefore required to avoid reaching ablative temperatures. In our experiments, we have set the maximum sonication power low enough to limit the temperature increase while the beam is focused at each cell of the grid during image acquisition (~ 2 s until a new temperature image is acquired). This could be further improved by continuously switching between several of foci (e.g. the three coldest cells), effectively spreading out the heat without the need for a faster image acquisition time. Diffused ultrasound focal fields [36] could also be implemented to the same effect. To avoid overheating at the foci during acquisition of monitoring images and in case of system malfunction, we have also implemented a safety feature that sets the power output to zero if no thermometry images in the control plane are received within 3 s.

Near field heating remains a problem due to the small active area and aperture of the endorectal transducer (23 × 40 mm), and the overlap or intersection of beam paths for the different foci. Our results showed excessive near-field heating for the 60 mm focal depth, which reduced to acceptable levels for the 35 mm focal depth. Therefore, with this transducer it may be difficult to achieve therapeutic temperatures localized at deep levels (greater than 40 mm) without incurring excessive temperatures in the near field. However, this depth limitation would be sufficient for HT treatments within prostate for a majority of patients. Based upon review of 800 prostate MRI exams performed at our institution, the mean anterior-posterior length of the gland was 3.4 cm, with the 95%ile = 4.7 cm, max = 7.2 cm (Susan Noworolski, Ph.D., personal communication). Pre-cooling the tissue with circulating water before and throughout the treatment helps to protect the rectum and tissue close to the transducer from over-heating and to move the effective treatment envelope away from the transducer, as was demonstrated in experiment 4. In vivo, blood perfusion is likely to lower the temperature elevation in the near field, as compared to the phantom experiments, but may also require more energy deposition to reach the target temperature. If precooling is used, the MR thermometry should cover the cooling period in order to accurately monitor the actual temperature change. Further development of this approach should include 3D temperature sampling, which would take into account temperature throughout the whole treated volume while controlling the ultrasound beam power and focusing.

As is the case with all phase difference based PRF thermometry methods, temperature errors due to motion, phase drift or susceptibility changes can occur. Due to the long temperature imaging duration for hyperthermia applications, phase drift could be a problem on some scanners. Several prospective or retrospective drift correction techniques, such as center frequency adjustment and post-acquisition correction as evaluated for hyperthermia by Bing et al. [37] can be applied to ameliorate the problem. In our study, correction for phase drift over less than 20-min duration was not necessary.

In MR thermometry, there is an inherent trade-off between temporal resolution and temperature uncertainty. MRgHT in general does not require fast imaging due to the diffuse heating and slow temperature rise [38]. On the other hand, the quick temperature rise in ablative procedures requires high temporal resolution (~ 1–3 s in non-moving organs). Similarly, accelerated MR thermometry was crucial for multi-focal rapid beam steering with feedback control, implemented in this project. Our acceleration parameters achieved 2 s update time using shared k-space with keyhole k-space sampling scheme. This resulted in a temperature uncertainty of 0.5 °C in our phantom experiments, which would be adequate to monitor hyperthermia within the prostate. In vivo, temperature uncertainty might be slightly higher, but could be improved by using multi-channel MR imaging coils.

To avoid image artifacts in thermometry frames following switching image orientations, dummy excitations with data acquisition disabled could be performed until magnetization reaches the steady state. Other acceleration techniques, such as echo-planar and non-Cartesian readouts, parallel imaging, and compressed sensing could allow for higher acceleration factors. Shared k-space with keyhole scheme was chosen since it was found to be robust to susceptibility artifacts near the focused ultrasound transducer. It did not require the use of multi-channel coils and allowed for minimal reconstruction delay. It is likely that a higher frame rate and/or more imaging slices could be achieved by further optimizing the keyhole width and the number of interleaves. The k-space sampling order could also be altered to acquire center lines last, ensuring more accurate temperature measurement at the time of reconstruction [39].

The main limitation of the current study is that phantom experiments do not precisely model the thermal and acoustic properties of in vivo tissue, such as tissue heterogeneity and the heat sink effect in tissue due to blood perfusion. Additional studies are necessary to assess the heating performance of this integrated system in in-vivo preclinical models.

## Conclusions

We have implemented a real-time MR thermometry-guided system for hyperthermia delivery within user-defined regions with the ExAblate prostate array and evaluated it in phantom experiments for different shapes and focal depths. Our results showed that uniform heating can be achieved and maintained at hyperthermic temperatures in the focal region. Near-field heating can be excessive for large focal depth but appears to be acceptable for geometries of normal sized prostates when precooling is applied. The shared k-space with keyhole acceleration pulse sequence acquired images with a 2 s update rate and provided a temperature accuracy of 0.5 °C, which was adequate for power and shape control during the HT experiments. Our results demonstrate the feasibility of using a commercially available endorectal FUS transducer to perform spatially-conformal hyperthermia therapy and could lead to a new set of exciting applications for these devices.

## Abbreviations

CEM43°C: Cumulative equivalent number of minutes at 43 °C
FUS: Focused ultrasound
HT: Hyperthermia therapy
MRgFUS: Magnetic resonance guided focused ultrasound
MRgHT: MR-guided hyperthermia therapy
MRI: Magnetic resonance imaging
PRFS: Proton resonance frequency shift
ROI: Region of interest
ROT: Region of treatment
SPGR: Spoiled gradient echo
